# Comparação entre Dois Escores de Risco quanto à Predição de Obstrução Microvascular Coronariana durante a Intervenção Percutânea Primária

**DOI:** 10.36660/abc.20200115

**Published:** 2021-05-06

**Authors:** Yuyang Xiao, Hua Chen, Dongxia Liu, Yanbo Wang, Wenlu Wang, Qian Zhang, Yuping Han, Xianghua Fu

**Affiliations:** 1 Hebei General Hospital Department of Cardiology Shijiazhuang China Hebei General Hospital - Department of Cardiology, Shijiazhuang, Hebei – China.; 2 Second Hospital of Hebei Medical University Department of Cardiology Shijiazhuang China Second Hospital of Hebei Medical University - Department of Cardiology, Shijiazhuang, Hebei – China.

**Keywords:** Infarto do Miocárdio, Intervenção Coronária Percutânea, Obstrução Coronariana, Previsões, Indicador de Risco

## Abstract

**Fundamento:**

Para pacientes com infarto do miocárdio com elevação do segmento ST (IAMCST) que sofrem de obstrução coronariana microvascular funcional e estrutural (OCM) subsequente, nenhuma abordagem terapêutica específica e definitiva de atenuação foi comprovada como válida em testes de larga escala atuais, o que destaca a necessidade de abordar seu reconhecimento precoce.

**Objetivos:**

Este estudo teve como objetivo comparar o desempenho de dois escores de risco clínico com uma medida objetiva de OCM durante intervenção coronária percutânea (ICP) em casos de IAMCST

**Métodos:**

A medição do índice de resistência microcirculatória (IRM) foi realizada e os parâmetros clínicos e angiográficos basais também foram registrados. Os pacientes foram divididos em entre os grupos OM (obstrução microvascular) e NOM (não-obstrução microvascular), de acordo com o valor de IRM pós-procedimento. O risco de OCM foi avaliado para todos os participantes pelos escores preditivos SAK e ATI, respectivamente. Cada sistema foi calculado somando-se as pontuações de todas as variáveis. As curvas de características do operador receptor (ROC) e a área sob a curva (AUC) de dois modelos de risco foram utilizadas para avaliar o desempenho discriminatório. Um ecocardiograma foi realizado sete dias após o procedimento para avaliar a fração de ejeção do ventrículo esquerdo (FEVE). Um valor P bicaudal de <0,05 foi considerado estatisticamente significativo.

**Resultados:**

Entre os 65 pacientes elegíveis com IAMCST, 48 foram alocados no grupo NOM e 17 no grupo OM, com uma incidência de OCM de 26,15%. Não houve diferença significativa na AUC entre os dois escores. A FEVE avaliada para o grupo NOM foi maior do que para o grupo OM.

**Conclusão:**

Os escores SAK e ATI tiveram bom desempenho para estimar o risco de OCM após ICP primário para pacientes com IAMCST.

## Introdução

Para pacientes com infarto agudo do miocárdio com elevação do segmento ST (IAMCST), a reperfusão oportuna da artéria relacionada ao infarto (ARI) já se mostrou a estratégia padrão-ouro para salvar o miocárdio isquêmico e inibir a remodelação ventricular. Durante o procedimento de recanalização da artéria origem, independentemente da patência do enxerto angiográfico, muitos pacientes desenvolvem perfusão insuficiente no tecido miocárdico decorrente de obstrução coronariana microvascular estrutural e funcional (OCM) no período perioperatório.^1^ A OCM, que é um reflexo de lesão microvascular persistente e foi anteriormente entendida como “fenômeno de no-reflow” (FNR), mostrou estar diretamente associada à extensão da área infartada e eventos cardiovasculares que aumentam e pioram o prognóstico de curto e/ou longo prazo.^2^^,^^3^ No entanto, para pacientes com IAMCST que sofrem de OCM subsequente, nenhuma abordagem terapêutica específica e definitiva de atenuação foi válida nos testes de grande escala atuais, o que destaca a necessidade de abordar o reconhecimento precoce e o pré-tratamento de pacientes de alto risco.

Recentemente, com base em alguns experimentos com animais e pesquisas clínicas, o mecanismo subjacente da OCM em um cenário de IAMCST agudo foi explorado. Embora a fisiopatologia exata não esteja clara, vários mecanismos, incluindo lesão de isquemia/reperfusão, embolização distal e suscetibilidade individual, são considerados responsáveis pela deterioração da perfusão microvascular integralmente.^4^ Consequentemente, apesar do fato de que numerosos ensaios sobre os possíveis fatores de influência de OCM ou FNR foram conduzidos, um único indicador pode não ser preciso o suficiente para avaliar o estado de perfusão da microvasculatura. Com base nessa suposição, desenvolvemos o modelo de risco SAK, composto por seis elementos independentes, incluindo tempo de início dos sintomas ao balão (SO-B), nível de ACT na admissão, classificação de Killip, idade, relação neutrófilos/linfócitos (RNL) e glicose. O modelo demonstrou bom desempenho preditivo para risco de OCM. Outros modelos de previsão também foram recentemente introduzidos por vários centros, com diferentes variáveis e conclusões. Dentre eles, o escore ATI pôde avaliar o comprometimento microvascular coronariano durante a intervenção coronária percutânea primária (ICP), na qual o IRM foi um parâmetro essencial.^5^

Desde 2003, o índice de resistência microcirculatória (IRM) tem sido descrito e aplicado gradativamente como um novo parâmetro invasivo do fluxo microvascular coronariano.^6^ Comparado com outros métodos não invasivos ou invasivos, o IRM tem as vantagens da boa reprodutibilidade, especificidade e independência de estenose epicárdica e dinâmica. Portanto, adotamos o índice como principal meio de avaliação da microvasculatura neste estudo. O objetivo deste artigo foi comparar o desempenho de previsão dos escores ATI e SAK para risco de OCM durante a ICP.

## Métodos

### Seleção de Pacientes

Neste estudo prospectivo, os candidatos admitidos no Departamento de Cardiologia do Segundo Hospital da Universidade Médica de Hebei de janeiro de 2018 a abril de 2018 foram inscritos consecutivamente. Todos os participantes preencheram os seguintes critérios: (1) ser diagnosticado com IAMCST de acordo com o padrão recomendados (sintomas típicos de dor no peito com duração de mais de 30 minutos sem alívio, segmento ST elevado a 0,1 mV em pelo menos duas derivações contínuas ou presumivelmente um novo bloqueio de ramo esquerdo (BRE) no exame eletrocardiográfico e valores aumentados de biomarcadores miocárdicos ou troponina cardíaca de alta sensibilidade positiva 7); (2) PIC primária agendada no laboratório de cateterismo de emergência 24 horas após o início da dor torácica até a admissão; e (3) ter concordado com o exame de IRM durante o procedimento. Os participantes que preencheram as seguintes características foram excluídos do estudo: (1) ter recebido agentes trombolíticos intravenosos; (2) ter sofrido choque cardíaco; (3) recusar o cateterismo primário ou ter uma intervenção seletiva agendada; (4) desenvolver dissecção ou complicações mecânicas durante o procedimento; (5) presença de múltiplas lesões adequadas para cirurgia de revascularização do miocárdio (CRM); (6) insuficiência hepática ou renal grave; (7) presença de tumor maligno; e (8) ter contraindicação à terapia antitrombótica e anticoagulante. O protocolo do estudo foi aprovado pelo comitê de ética local, de acordo com a Declaração de Helsinque. Todos os pacientes selecionados assinaram o termo de consentimento livre e esclarecido antes do estudo.

Na admissão, os históricos médicos dos pacientes foram imediata e brevemente coletados. Um eletrocardiograma de 18 derivações foi realizado nos primeiros 10 minutos. Todos os pacientes receberam prescrição de doses de ataque de Aspirina (300mg) e Ticagrelor (180mg) ao receberem os diagnósticos de IAMCST. Amostras de sangue venoso foram coletadas para exames laboratoriais, incluindo rotina de sangue, ensaio bioquímico [proteína C reativa de alta sensibilidade (PCR-as), função hepática e renal, glicose, lipídios, eletrólitos], biomarcadores miocárdicos [creatina quinase e isoenzima creatinoquinase MB (CK, CK-MB)], troponina I cardíaca (cTnI), D-dímero, peptídeo natriurético cerebral de plasma (BNP) e tempo de coagulação ativado (TCA). O teste de TCA foi realizado com êmbolo mecânico de dois canais (ACT plus, Medtronic Inc., Minneapolis, Minnesota, EUA) com temperatura de reação de 37 ºC. Todos os participantes assinaram o termo de consentimento livre e esclarecido antes da operação.

### Tratamento e Avaliação

O procedimento foi realizado de acordo com a prática clínica padrão via acesso radial, ulnar ou femoral. A revisão e a análise angiográfica foram realizadas por pelo menos dois cardiologistas intervencionistas qualificados. A gravidade da estenose da artéria coronária foi medida usando o sistema Quantitative Coronary Analysis (QCA). Se o grau da ARI fosse superior a 75%, o implante de stent farmacológico foi considerado uma terapia de reperfusão primária útil. Os pacientes receberam heparina não fracionada intravenosa (HNF) 70-100U/kg para manter os níveis de ACT de 250-300 segundos convencionalmente, enquanto a bivalirudina serviu como alternativa se os pacientes apresentassem alto risco de hemorragia. As doses do anticoagulante foram ajustadas com base nas condições individuais dos pacientes e na aplicação do inibidor da glicoproteína (Tirofiban). Dispositivos de rotina (stents, balões, cateteres e fios), procedimentos intervencionistas (os números e a pressão de pré-dilatação e pós-dilatação, aspiração de trombo e implante de marcapasso temporário) e medicação adjuvante foram determinados pelos operadores.

Os dados do tempo de reperfusão, incluindo o início dos sintomas até a introdução do balão (SO-B), o primeiro contato médico até o procedimento com balão (FMC-B) e o grau de fluxo inicial da trombólise no infarto do miocárdio (TIMI)^8^ da artéria originária foram cuidadosamente avaliados e registrados. Assim que o fio-guia cruzou ou o balão inflou as lesões originárias, a carga de trombo da ARI foi analisada e avaliada.^9^ Após a revascularização, o grau de fluxo da TIMI, o grau de perfusão miocárdica TIMI (GPTIMI)^10^ e a contagem de quadros TIMI corrigida (cTFC) da artéria foram avaliados, conforme descrito anteriormente. O cTFC da artéria originária foi contado a uma taxa de 15 quadros por segundo, de acordo com o método de Gibson.^11^ Todos os pacientes inscritos receberam terapia anticoagulante e antitrombótica, estatinas, bloqueador do receptor β, inibidores da enzima de conversão da angiotensina/bloqueador do receptor da angiotensina e/ou nitratos, de acordo com as diretrizes mais recentes.

Após a insuflação do balão, todos os pacientes foram submetidos a IRM mediante colocação de stent com o cabo de pressão (Pressure Wire Certus, C12008, St. Jude Medical System AB, Uppsala, Suécia). O fio e o sensor de pressão/temperatura foram colocados na extremidade distal do vaso. Após a calibração do aparelho, 3 mL de solução salina em temperatura ambiente foram injetados três vezes no cateter-guia para a coleta dos dados basais. O trifosfato de adenosina dissódico foi administrado por transfusão intravenosa a uma velocidade de 140*μ*g/kg·min para atingir hiperemia coronariana. O tempo de trânsito médio hiperêmico (Tmn-Hyp) foi obtido por injeção repetida de solução salina. O valor da pressão da artéria distante (Pd) exibido na tela foi cuidadosamente registrado e o fio de pressão permaneceu na mesma posição durante a avaliação do IRM para garantir a confiabilidade do resultado. Após a implantação do stent, o valor IRM da artéria originária foi medido novamente para estimar o status de perfusão miocárdica. Os valores de IRM pré e pós-intervenção foram calculados usando a seguinte fórmula, sem considerar o aumento da pressão coronária:

$$\text{IMR}=\text{Pd}\times \text{Tmn}-{\text{Hyp}}^{6}$$

Todos os participantes receberam números de identificação de acordo com a ordem cronológica da operação e foram alocados em grupos diferentes de acordo com os valores IRM finais após a intervenção: grupo NOM (Não obstrução microvascular), com valores IRM superiores a 40 U, e grupo OM (obstrução microvascular), com valores de IRM não superiores a 40 U. O risco de OCM foi avaliado por meio de dois modelos de risco, sendo que cada pontuação foi calculada somando-se os pontos de todas as variáveis. Os detalhes do escore SAK estão apresentados na Tabela 1 e do ATI, na Tabela 2.

**Tabela 1:** Escore SAK

Idade	Pontos	SO-B (hrs)	Pontos	TCA	Pontos	Killip	Pontos	RNL	Pontos	GLI	Pontos
≤65	0	0-1	1	≤ 60	9	I	0	≤7,0	0	≤12,0	0
>65	2	1-2	2	60-80	8	I I	4	>7,0	4	>12,0	2
		2-3	3	80-100	7	I I I	8				
		3-4	4	100-120	6						
		4-5	5	120-140	5						
		…		140-160	4						
		20-21	21	160-180	3						
		21-22	22	180-200	2						
		22-23	23	200-220	1						
		23-24	24	>220	0						

SO-B: sintomas ao balão; TCA: tempo de coagulação ativado; RNL: relação neutrófilos/linfócitos; GLI: Glicose.

**Tabela 2:** Escore ATI

Idade	Pontos	Pontuação do trombo	Pontos	IRM-pré	Pontos
≤50	0	0-3	0	< 40	0
>50	1	4	1	40-100	1
		5	3	>100	2

IRM: índice de resistência microcirculatória.

Uma ecocardiografia transtorácica bidimensional foi realizada sete dias após o procedimento em todos os pacientes para avaliação da função ventricular esquerda e remodelação.

### Análise Estatística

A análise estatística foi realizada no software SPSS (Versão 23.0, SPSS Inc., Chicago, Illinois, EUA). Variáveis contínuas foram testadas para distribuição normal com o teste de Kolmogorov-Smirnov. Os dados normalmente distribuídos foram apresentados em média ± desvio padrão (DP) e comparados pelo teste t de Student entre os grupos. Os dados com distribuição não normal foram apresentados em mediana (primeiro quartil, terceiro quartil) e comparados pelo teste U de Mann-Whitney. Variáveis categóricas foram expressas em porcentagem e comparadas por meio do teste qui-quadrado ou do teste exato de Fisher. O desempenho discriminatório do modelo construído foi examinado pela curva Característica de Operação do Receptor (ROC). A ilustração da curva ROC dos escores foi feita no Software MedCalc (Versão 15.2.2, Med Calc Software bvba, Ostend, Bélgica). A área sob a curva (AUC), o valor de corte, sensibilidade, a especificidade e o Índice de Youden correspondente de cada curva ROC foram então obtidos (Índice de Youden = sensibilidade + especificidade -1). Os escores foram comparados por meio de um teste não paramétrico. O valor de p bicaudal de <0,05 foi considerado estatisticamente significativo.

## Resultados

### Alocação nos Grupos

De janeiro de 2018 a abril de 2018, um total de 65 pacientes elegíveis com IAMCST foram incluídos neste estudo. Com base no limiar IRM final de 40, alocamos 48 pacientes no grupo NOM e 17 no grupo OM, com uma incidência de OCM de 26,15%.

### Características Clínicas de Base

A comparação dos dados demográficos, características clínicas basais e exames laboratoriais pré-operatórios entre os grupos estão na Tabela 3. Não foi observada diferença significativa nos seguintes parâmetros: sexo, índice de massa corporal (IMC), sinais vitais, história prévia, contagem de hemácias, contagem de plaquetas, proteína C reativa de alta sensibilidade (PCR-as), eletrólitos e lipídios (todos p>0,05). A média de idade do grupo OM foi maior do que a do grupo NOM (p = 0,002). Os pacientes do grupo OM compartilharam uma proporção maior da classificação de Killip 3 e uma proporção menor da Killip 1. Os escores GRACE e CRUSADE também foram significativamente maiores no grupo OM. Houve diferenças estatísticas nos seguintes itens laboratoriais entre os grupos: contagem de leucócitos, contagem de neutrófilos, contagem de linfócitos, razão neutrófilos/linfócitos (RNL), CK-MB, cTNI, ACT, creatinina sérica, eGFR, glicose, D-dímero e BNP (Todos p <0,05).

**Tabela 3:** Características clínicas basais entre os grupos

Variáveis		Grupo NOM (n=48)	Grupo OM (n=17)	Valor p
Idade (anos)		56,51±8,99	64,96±9,43	0,002
Masculino, n (%)		42(87,50)	13(76,47)	0,434
IMC (kg/m^2^)		24,76±3,31	25,52±3,12	0,412
Pressão arterial sistólica (mmHg)		128,54±19,30	136,67±22,49	0,158
Pressão arterial diastólica (mmHg)		79,03±10,22	75,41±14,80	0,271
Frequência cardíaca (bpm)		74,28±18,69	77,35±16,65	0,552
**Classificação Killip**				
	Classe I, n (%)	29(60,42)	4(23,53)	0,009
	Classe II, n (%)	16(33,33)	4(23,53)	0,452
	Classe III, n (%)	3(6,25)	9(52,94)	<0,001
Histórico de DAC, n (%)		25(52,08)	11(64,71)	0,368
Hipertensão, n (%)		27(56,25)	10(58,82)	0,854
Diabetes, n (%)		15(31,25)	9(52,94)	0,111
Hiperlipidemia, n (%)		22(45,83)	8(47,06)	0,931
Fumantes, n (%)		18(37,50)	9(52,94)	0,267
**Teste laboratorial na admissão**				
	Contagem de leucócitos (10^9^/ L)	9,84±2,51	12,45±2,89	<0,001
	Contagem de neutrófilos (10^9^/ L)	7,63(6,18, 9,09)	11,65(10,18, 13,00)	<0,001
	Contagem de linfócitos (10^9^/ L)	1,60(1,26, 2,00)	1,46(1,08, 1,70)	0,184
Razão N/L		4,95(3,85, 7,00)	9,52(6,98, 10,56)	<0,001
PCR-as (mg/L)		4,10(2,10, 6,55)	4,30(2,95, 7,30)	0,565
TCA		154(135, 178)	105(88, 132)	<0,001
CK-MB (U/L)		111 (43, 251)	168(84, 335)	0,044
Troponina cardíaca I (ng/mL)		3,5 (1,8, 8,9)	14,0(6,0, 28,5)	<0,001
Creatinina sérica (μmol/L)		77,50(71,35, 86,15)	87,8 (77,5, 93,73)	0,038
TFG (mL/min/1,73m^2^)		98,70±14,62	85,89±17,08	0,004
Potássio sérico (mmol/L)		3,81±0,55	3,83±0,43	0,886
colesterol LDL (mmol/L)		2,87±0,67	2,80±0,83	0,717
Glicose (mmol/L)		8,57±1,88	11,31±2,41	<0,001
Dímero-D (μg/mL)		0,14(0,10, 0,23)	0,25 (0,16, 0,50)	<0,001
BNP (pg/mL)		50(26,150)	190(78,420)	0,003
**Medicação pré-procedimento**				
Terapia antiplaquetária dupla, n (%)		48(100,00)	15(88,24)	0,065
Estatinas, n (%)		24(50,00)	7(41,18)	0,531
Bloqueador beta, n (%)		3(6,25)	2(11,76)	0,6
Escore GRACE		137,48±23,91	152,94±27,97	0,032
Escore CRUSADE		22,75±12,34	29,77±12,29	0,045

NOM: não-obstrução microvascular; IMC: índice de massa corporal; DAC: doença arterial coronariana; PCR-as: proteína C reativa de alta sensibilidade; TCA: tempo de coagulação ativado; TFG: taxa de filtração glomerular; BNP: peptídeo natriurético do tipo B.

### Análise Angiográfica e Medição Invasiva de Perfusão Microvascular

As características angiográficas de todos os participantes estão resumidas na Tabela 4. O tempo SO-B do grupo OM foi aparentemente atrasado em comparação com o do grupo NOM (p = 0,002), sendo que não houve diferença significativa no tempo de FMC para o FMC-B (p = 0,843). Após a intervenção, uma diferença significativa em relação aos indicadores de perfusão do fluxo sanguíneo foi observada, incluindo a proporção do grau TIMI 3 (p<0,001), cTFC (p<0,001) e a proporção de GPTIMI 3 (p<0,001). Outras informações angiográficas e de procedimento, como distribuição da ARI, detalhes de stent, medicação, tratamento suplementar e volume de meio de contraste foram comparáveis entre os grupos (todos p>0,05).

**Tabela 4:** Características procedurais e angiográficas entre os grupos

Variáveis		Grupo NOM (n=48)	Grupo OM (n=17)	Valor p
Início até o balão (hours)		4,0(3,0; 5,0)	6,5(5,0; 12,0)	0,002
FMC até o balão (horas)		2,0(1,0; 3,0)	1,5(1,0; 2,8)	0,843
Parede miocárdica, n (%)				
	Parede anterior	19(44,19)	9(52,94)	0,339
	Outros	29(55,81)	8(47,06)	0,339
**Número de artérias estenosadas, n (%)**				
1		9(18,75)	4(23,53)	0,729
	2	18(37,50)	7(41,18)	0,789
	3	21 (43,75)	6(35,29)	0,543
**Fluxo TIMI inicial, n (%)**				
	0	27(56,25)	14(82,35)	0,055
	1	8(16,67)	2(11,76)	1
	2	8(16,67)	1(5,89)	0,426
	3	5(10,41)	0(0,00)	0,315
**Pontuação de trombo, n (%)**				
	0-3	24(50,00)	1(5,56)	0,001
	4	20(41,67)	7(41,18)	0,972
	5	4(8,33)	9(53,26)	<0,001
**Fluxo TIMI final, n (%)**				
	0	0(0,00)	1(5,88)	0,262
	1	0(0,00)	3(17,65)	0,016
	2	0(0,00)	11(64,71)	<0,001
	3	48(100,00)	2(11,76)	<0,001
ARI-cTFC		24(20; 32)	48(36; 58)	<0,001
**GPTIMI, n (%)**				
	0	0(0,00)	2(11,76)	<0,001
	1	0(0,00)	5(29,41)	<0,001
	2	5(10,42)	11(58,83)	<0,001
	3	43(89,58)	0(0,00)	<0,001
**IRM-pré**				
	< 40	16(33,33)	1(5,58)	0,029
	40-100	20(41,67)	5(29,41)	0,372
	>100	12(25,00)	11(64,71)	0,003
**Número do stent por paciente, n (%)**				
1		42(87,50)	12(70,59)	0,138
	≤2	6(12,50)	5(29,41)	0,138
Comprimento do stent (mm)		23(21; 28)	24(18; 31)	0,143
Diâmetro do stent (mm)		2,25(2,20; 3,00)	2,50(2,25; 3,00)	0,859
Pressão de pré-dilatação (atm)		14(12; 16)	14(12; 15)	0,307
Números de pré-dilatação		3(2; 5)	4(3; 5)	0,422
Pressão de expansão do stent (atm)		14(14; 16)	14(12; 16)	0,347
Pressão pós-dilatação (atm)		16(12; 17)	14(11; 16)	0,776
Números pós-dilatação		2(2; 3)	2(1; 3)	0,689
Aspiração de trombo, n (%)		12(25,00)	3(17,64)	0,741
Marcapasso temporário, n (%)		4(8,33)	1(5,88)	1
Circulação colateral, n (%)		9(18,75)	3(17,65)	1
Volume do meio de contraste (mL)		160(140; 190)	180(150; 210)	0,06
**Medicação de procedimento, n (%)**				
	Tirofiban	43(89,58)	14(82,35)	0,421
	Bivalirudina	9(18,75)	5(29,41)	0,493
	Anisodamina	8(16,67)	3(17,65)	1

NOM: não-obstrução microvascular; OM: obstrução microvascular; TIMI: trombólise no infarto do miocárdio; GPTIMI: grau de perfusão miocárdica TIMI; IRM: índice de resistência microcirculatória

### Curva ROC dos Dois Escores e Comparação de AUC

As pontuações correspondentes dos dois sistemas foram calculadas para todos os participantes. Com base nas pontuações e incidência de OCM, a curva ROC foi traçada. Para os escores SAK, a AUC foi de 0,855 [intervalo de confiança (IC) de 95%: 0,746 - 0,930], com um valor de corte de 15 e um índice de Youden de 0,6078. Para o escore ATI, a AUC foi de 0,907 (IC95%: 0,809 - 0,965), com valor de corte de 3 e Índice de Youden de 0,6875. Não houve diferença significativa na AUC (Z = 1,001, p = 0,317) (Tabela 5).

**Tabela 5:** Comparação de AUC e detalhes relacionados dos escores SAK e ATI

Variáveis	AUC	IC95%	Ponto de corte	Índice Youden	Z	p
Escore SAK	0,855	0,746 - 0,930	15	0,6078	1,001	0,317
Escore ATI	0,907	0,809 - 0,965	3	0,6875		

### Ecocardiografia

Todos os pacientes aceitaram fazer a ecocardiografia transtorácica após o procedimento no hospital. A fração de ejeção do ventrículo esquerdo (FEVE) do grupo NOM foi maior do que a do grupo OM (56,03 ± 5,22 vs. 47,79 ± 6,38, p <0,001).

## Discussão

Apesar do grande progresso alcançado nas estratégias terapêuticas do infarto do miocárdio nas últimas décadas, o comprometimento microvascular continua sendo uma questão importante durante o cateterismo primário. Estima-se que a reperfusão insuficiente no nível do tecido miocárdico possa chegar a 50% dos casos, apesar do sucesso da recanalização epicárdica.^12^ Os benefícios decorrentes das estratégias de reperfusão farmacêutica ou mecânica estariam comprometidos na presença de obstrução coronariana microvascular, que está associada à função cardíaca deficiente e desfechos desfavoráveis.

Devido à falta de tratamento específico e atenuação da OCM, o reconhecimento precoce e o pré-tratamento de pacientes de alto risco são de grande importância. Os indicadores de identificação foram examinados intensamente por consideráveis estudos anteriores. No entanto, considerando que se acredita que um grande número de mecanismos complicados contribua para o desenvolvimento da obstrução microvascular, um único elemento pode não ser convincente o suficiente na avaliação da predição e estratificação de risco. Portanto, a avaliação de sistemas compostos por vários índices para avaliar a probabilidade dessa complicação proporciona melhor detecção e diagnóstico. Além dos dois modelos analisados neste estudo, escores de FNR também foram previamente desenvolvidos.

Dogan et al.,^13^ relatou que a hiperglicemia, o tempo isquêmico prolongado e a baixa contagem de neutrófilos são atribuídos ao desenvolvimento do modelo de risco.^13^ Bayramogluet al.,^14^ construiu o modelo preditivo abrangendo idade, valor da FEVE, pontuação SYNTAX, comprimento do stent, pontuação da carga do trombo, classificação de Killip e tempo de reperfusão.^14^ O estudo retrospectivo conduzido por Wang et al.,^15^ também mostrou que idade, tempo da dor até ICP, contagem de neutrófilos, nível de glicose na admissão, pontuação de trombo pré-ICP, circulação colateral e classificação Killip poderiam ser adotados para estabelecer o modelo de no-reflow.^15^ Devido aos diferentes protocolos de estudo, tamanho da amostra, não foram obtidas medidas auxiliares e conclusões consistentes.

Em vez dos padrões angiográficos (fluxo sanguíneo TIMI, GPTIMI ou graus de blush miocárdico) aplicados nos ensaios clínicos anteriores, o IRM foi introduzido para determinar a perfusão da microcirculação no presente estudo. O IRM, uma medida quantitativa derivada da termodiluição da função microvascular coronariana, foi proposto pela primeira vez por Fearon em 2003. O modelo Porcine também foi usado para investigar a correlação entre o valor do IRM calculado e a resistência distal verdadeira, validando a viabilidade desta técnica inovadora para estimar a resistência microvascular.^6^ Diferente de outras avaliações angiograficamente fisiológicas e funcionais, o IRM compartilha as vantagens de independência da estenose epicárdica, reprodutibilidade superior e instabilidade hemodinâmica. Bulluck revisou a literatura e relatou que um limiar pós-procedimento de 40 U era válido na identificação de OCM para aqueles que realizaram medições de IRM.^16^

Além de estar diretamente relacionado ao estado de perfusão do tecido miocárdico, o IRM também mostrou ter uma forte associação com os níveis de pico de creatina quinase, prognóstico do paciente e recuperação do desempenho ventricular no contexto de IAMCST,^17^^–^^20^ que lançou as bases para desenvolvimento do ATI. O escore ATI foi introduzido pela primeira vez por De Maria et al. consistindo principalmente de três características, incluindo idade, escore de trombo e valor de IRM pré-stent.,^5^ O escore ATI também foi considerado uma ferramenta promissora para prever reperfusão miocárdica subótima em pacientes com IAMCST e está correlacionado com a área de infarto medida por ressonância magnética (RM) cardíaca em estudos subsequentes.^21^

Limitada por custos e regulamentos de seguro relacionados, entretanto, o IRM nem sempre está disponível ou é aceitável nas práticas atuais. Também só poderia ser implementado no Laboratório de Cateterização emergente. Nesse sentido, com base nas evidências existentes e em nossa experiência prática, rastreamos sistematicamente as possíveis informações clínicas e angiográficas, desenvolvendo escores preditivos SAK que incorporam seis variáveis convencionais: idade, classificação de Killip, início dos sintomas ao tempo do balão, níveis iniciais de TCA, RNL e valores de glicose. Nosso estudo anterior verificou sua capacidade e eficácia na avaliação de pacientes com alto risco de OCM.^22^ Portanto, tentamos comparar o desempenho dos escores SAK e ATI na previsão do risco potencial de microvasculatura prejudicada durante a intervenção primária, auxiliando os médicos no pré-tratamento imediato para minimizar a incidência desta condição antes que o procedimento ocorra.

Visivelmente, um valor de AUC ou índice C acima de 0,75 em um modelo desenvolvido é reconhecidamente uma validação confiável. A partir dos resultados obtidos, a AUC dos escores SAK e ATI foram de 0,855 e 0,907, respectivamente, o que provou que ambos os sistemas de estimativa foram capazes de prever o risco potencial de OCM e tiveram um bom desempenho. A AUC do escore ATI parecia mais alta, mas não houve diferença óbvia no desempenho para a avaliação de risco.

Embora o escore ATI tivesse um desempenho favorável para previsão, havia algumas diferenças em comparação com o relatório original de desenvolvimento da ATI. Em primeiro lugar, o padrão de pontuação de trombo mais comumente escolhido foi estabelecido por Gibson. No entanto, de acordo com a prática clínica e dados anteriores, apenas 0,4% dos casos tiveram pontuação 5 após o fio-guia ou balão passar pelas lesões ocluídas, enquanto quase 30% dos casos compartilharam uma pontuação de 4.^23^ Consequentemente, a pontuação da carga do trombo foi avaliada após a passagem do fio-guia ou insuflação do balão. Em segundo lugar, o valor de pico dos biomarcadores miocárdicos e da troponina cardíaca não foram documentados como no estudo original, levando-se em consideração a ecocardiografia e que a diferença da FEVE também foi precisa o suficiente para mostrar a relação entre a perfusão da microcirculação e a área do infarto. A partir dos resultados da ecocardiografia, pudemos deduzir que os pacientes com OCM apresentavam pior função cardíaca, o que era consistente com as evidências existentes, enfatizando a importância particular de melhorar o estado de perfusão da circulação microvascular.^24^

Apesar de suas vantagens, o IRM não está disponível ou é aplicável na maioria dos hospitais locais e muitos pacientes recusam esse exame devido ao seu custo. Da mesma forma, um escore SAK consistindo em índices comuns, atualmente, parecia ser uma alternativa no campo clínico.

Este estudo, entretanto, tem algumas limitações. Primeiro, foi um estudo de centro único com amostra de tamanho relativamente pequeno. Os escores de risco foram validados pelas informações de um banco de dados de centro único. O poder discriminatório dos modelos requer uma investigação e validação de escala amostral maior. Em segundo lugar, o TCA era um elemento essencial no escore SAK, enquanto o nível de TCA é influenciado por uma série de fatores na prática; portanto, o intervalo de referência na pontuação pode ser diferente dependendo da equipe de teste e do equipamento. Terceiro, os pacientes com choque cardíaco não foram inscritos, pois pode ser necessário tratamento suplementar de suporte à vida e as características basais seriam desequilibradas para esses sujeitos.

## Conclusão

Neste estudo, nossos dados mostraram que os escores SAK e ATI tiveram um bom desempenho na estimativa do risco de OCM após o ICP primário para pacientes com IAMCST agudo. Portanto, esses escores são precisos para prever OCM quando comparados às medidas invasivas obtidas no IRM.
