# Supplementary material for: Comparação entre Dois Escores de Risco quanto à Predição de Obstrução Microvascular Coronariana durante a Intervenção Percutânea Primária
Source: Arq Bras Cardiol. 2021 May 6;116(5):959–67. [Article in Portuguese] doi: 10.36660/abc.20200115 (PMC8121479; doi:10.36660/abc.20200115)
Supplement: Translation

# Translation: The Comparison between Two Risk Scores as for the Prediction of Coronary Microvascular Obstruction during Primary Percutaneous Intervention

**Authors:** Yuyang Xiao, Hua Chen, Dongxia Liu, Yanbo Wang, Wenlu Wang, Qian Zhang, Yuping Han, Xianghua Fu

**Affiliations:** 1 Shijiazhuang China Hebei General Hospital - Department of Cardiology, Shijiazhuang, Hebei – China.; 2 Shijiazhuang China Second Hospital of Hebei Medical University - Department of Cardiology, Shijiazhuang, Hebei – China.

**Keywords:** Myocardial Infarction, Percutaneous Coronary Intervention, Coronary Obstruction, Forecasting, Risk Index

## Abstract

**Background:**

For patients with ST-segment elevation myocardial infarction (STEMI) that are suffering from subsequent coronary microvascular functional and structural obstruction (CMVO), no specific and definitive therapeutic approaches of attenuation have been proven valid in up-to-date large-scale tests, which highlights the urge to address its early recognition.

**Objectives:**

This study aimed to compare the performance of two clinical risk scores with an objective measurement of CMVO during percutaneous coronary intervention (PCI) with STEMI.

**Methods:**

The Index of Microcirculatory Resistance (IMR) measurement was conducted and the baseline clinical and angiographic parameters were also recorded. The patients were divided into MO (Microvascular obstruction) or NMO (Non-microvascular obstruction) groups according to the post-procedure IMR value. The CMVO risk was evaluated for all participants by SAK and ATI predictive scores, respectively. Each system was calculated by summing the scores of all variables. The receiver operator characteristic (ROC) curves and the area under the curve (AUC) of two risk models were used to evaluate the discriminatory performance. An echocardiography was performed seven days after the procedure to evaluate left ventricular ejection fraction (LVEF). A two-sided P-value of <0.05 was considered statistically significant.

**Results:**

Among the 65 eligible STEMI patients, 48 patients were allocated in the NMO group and 17 in the MO group, with a CMVO incidence of 26.15%. There was no significant difference in the AUC between both scores. The LVEF evaluated for the NMO group was higher than that of MO group.

**Conclusion:**

Both SAK and ATI scores performed well in estimating CMVO risk after primary PCI for STEMI patients.

## Introduction

For patients with acute ST-segment elevation myocardial infarction (STEMI), the timely reperfusion of the infarct-related artery (IRA) has been shown to be the gold-standard strategy to save the ischemic myocardium and inhibit ventricular remodeling. During the recanalization procedure of the culprit artery, regardless of angiographic grafting patency, many patients develop insufficient perfusion in the myocardial tissue resulting from coronary microvascular functional and structural obstruction (CMVO) in the perioperative period.^1^ CMVO, which is a reflection of persistent microvascular injury and has been previously understood as the “no-reflow phenomenon” (NRF), was previously shown to be directly associated with infarcted area extension and cardiovascular events that increase and worsen patients’ short and/or long-term prognosis.^2^^,^^3^ Nevertheless, for STEMI patients suffering from subsequent CMVO, no specific and definitive therapeutic approaches of attenuation have been valid in the present large-scale tests, which highlights the urge to address early recognition and the pretreatment of high-risk patients.

Recently, based on some animal experiments and clinical research, the underlying mechanism of CMVO in an acute STEMI setting has been explored. Although the exact pathophysiology is unclear, multiple mechanisms including ischemia/reperfusion injury, distal embolization, and individual susceptibility are assumed to be responsible for deteriorating microvascular perfusion integrally.^4^ Accordingly, despite the fact that numerous trials on the possible influencing factors of CMVO or NRF have been conducted, one single indicator might not be accurate enough in evaluating the perfusion state of the microvasculature. Based on this assumption, we have developed the SAK risk model, composed of six independent elements, including symptom onset to balloon (SO-B) time, admission activated clotting time (ACT) level, Killip classification, age, neutrophil/lymphocyte ratio (NLR), and glucose value. The model was proven to have good predictive performance for CMVO risk. Other predicting models have also been recently introduced by various centers, with different variables and conclusions. Among them, the ATI score was capable of evaluating coronary microvascular impairment during primary percutaneous coronary intervention (PCI), in which IMR was an essential parameter.^5^

Since 2003, the index of microcirculatory resistance (IMR) has been described and applied gradually as a new invasive parameter of the coronary microvascular flow.^6^ Compared with other noninvasive or invasive methods, IMR has the advantages of good reproducibility, specificity, and independence of epicardial stenosis and dynamics. Therefore, we adopted the IMR as the main means of assessment of the microvasculature in this study. The aim of this paper was to compare the predicting performance of the ATI and SAK scores for CMVO risk during PCI.

## Methods

### Patient Selection

In this prospective study, candidates admitted to the Cardiology Department of the Second Hospital of Hebei Medical University from January 2018 to April 2018 were enrolled consecutively. All participants met the following criteria: (1) being diagnosed with STEMI according to the guide-recommended standard (typical chest pain symptoms lasting more than 30 minutes without relief, ST-segment elevated 0.1 mV in at least two continuous leads or presumably new left bundle branch block (LBBB) on electrocardiographic examination and increased myocardial biomarker values or positive high-sensitive cardiac troponin)^7^; (2) being scheduled for primary PCI in the emergent catheterization laboratory 24 hours after chest pain onset to admission; and (3) having agreed with IMR examination during the procedure. The participants who met the following features were excluded from the study: (1) having received intravenous thrombolytic agents; (2) having had experienced cardiac shock; (3) refusing primary catheterization or having a selective intervention planned; (4) developing dissection or mechanical complications during procedure; (5) presence of multiple lesions suitable for coronary artery bypass grafting (CABG); (6) presence of severe hepatic or renal insufficiency; (7) Presence of a malignant tumor; and (8) having a contraindication to antithrombotic and anticoagulation therapy. The study protocol was approved by the local ethics committee, as per the Helsinki Declaration. All the selected patients signed an informed consent form prior to the study.

Upon admission, the patients’ brief medical histories were immediately taken. An 18-lead electrocardiogram was performed within 10 minutes. All patients were prescribed with loading doses of Aspirin (300mg) and Ticagrelor (180mg) upon receiving the STEMI diagnoses. Venous blood samples were collected for laboratory testing, including blood routine, biochemical assay [high-sensitivity C-reactive protein (hs-CRP), hepatic and renal function, glucose, lipid, electrolyte], myocardial biomarkers [creatine kinase and its MB isozyme (CK, CK - MB)], cardiac troponin I (cTnI), D-Dimer, plasma brain natriuretic peptide (BNP), and ACT. The ACT test was performed with a two-channel mechanical plunger (ACT plus, Medtronic Inc., Minneapolis, Minnesota, USA) with reaction temperature of 37ºC. All participants signed an informed consent form prior to the operation.

### Treatment and Evaluation

The interventional procedure was performed according to the standard clinical practice via radial, ulnar or femoral access. The angiographic review and analysis were accomplished by at least two qualified interventional cardiologists. The coronary artery stenosis severity was measured using the Quantitative Coronary Analysis (QCA) system. If the severity degree of the IRA was over 75%, drug-eluting stenting was considered a useful primary reperfusion therapy. The patients received intravenous unfractionated heparin (UFH) 70-100U/kg to maintain the ACT levels of 250-300 seconds conventionally, while Bivalirudin served as an alternative if patients had a high hemorrhage risk. Anticoagulant doses were adjusted based on the individual conditions of patients and on the application of the glycoprotein inhibitor (Tirofiban). Routine devices (stents, balloons, catheters, and wires), interventional procedures (the numbers and the pressure of pre-dilation and post-dilation, thrombus aspiration, and temporary pacemaker implantation), and adjuvant medication were determined by the operators. Reperfusion time data, including symptom onset to balloon time (SO-B) and first medical contact to balloon time (FMC-B) and the initial thrombolysis in myocardial infarction (TIMI) flow grade^8^ of the culprit artery were carefully assessed and recorded. As soon as the guidewire crossed or the balloon inflated the culprit lesions, the thrombus burden of the IRA was analyzed and scored.^9^ After revascularization, the TIMI flow grade, TIMI myocardial perfusion grade (TMPG),^10^ and corrected TIMI frame count (cTFC) of the artery were evaluated, as previously described. The culprit artery cTFC was counted at the rate of 15 frames per second, in accordance with the Gibson's method.^11^ All the enrolled patients received anticoagulant and antithrombotic therapy, statins, β receptor blocker, angiotensin-converting enzyme inhibitors/angiotensin receptor blocker, and/or nitrates according to the latest guidelines.

After balloon inflation, all patients were subjected to the IMR measurement upon stenting with the pressure wire (Pressure Wire Certus, C12008, St. Jude Medical System AB, Uppsala, Sweden). The wire and the pressure/temperature sensor on the head were placed on the distal end of the vessel. After the device calibration, 3 mL of saline at room temperature was injected three times through the guiding catheter to collect the baseline data. Adenosine disodium triphosphate was administered by intravenous transfusion at a speed of 140μg/ kg·min to achieve coronary hyperemia. The hyperemic mean transit time (Tmn-Hyp) was obtained by repeated saline injection. The value of the distant artery pressure (Pd) displayed on the screen was carefully recorded and the pressure wire remained in the same position during IMR assessment to guarantee the reliability of the result. After stent deployment, the IMR value of the culprit artery was measured again to estimate the myocardial perfusion status. Pre- and post-intervention IMR values were calculated using the following formula, without considering the coronary wedge pressure:

$$\text{IMR}=\text{Pd}\times \text{Tmn}-{\text{Hyp}}^{6}$$

All participants were given ID numbers according to the operation chronological order and were assigned to different groups according to the final IMR values after the intervention, namely the NMO (Non-Microvascular Obstruction) group, with IMR values over 40 U, and the MO (Microvascular Obstruction) group, with IMR values of not more than 40 U. The CMVO risk was evaluated for those participants by two risk models, each score was calculated by summing the points of all variables. The details of the SAK score are presented in Table 1 and the ATI scores are listed in Table 2.

**Table 1:** SAK score

Age	Points	SO-B (hrs)	Points	ACT	Points	Killip	Points	NLR	Points	GLU	Points
≤65	0	0-1	1	≤ 60	9	I	0	≤7.0	0	≤12.0	0
>65	2	1-2	2	60-80	8	II	4	>7.0	4	>12.0	2
		2-3	3	80-100	7	III	8				
		3-4	4	100-120	6						
		4-5	5	120-140	5						
		…		140-160	4						
		20-21	21	160-180	3						
		21-22	22	180-200	2						
		22-23	23	200-220	1						
		23-24	24	>220	0						

ACT: activated clotting time; NLR: neutrophil/lymphocyte ratio; GLU: glucose.

**Table 2:** ATI score

Age	Points	Thrombus Score	Points	IMR-pre	Points
≤50	0	0-3	0	< 40	0
>50	1	4	1	40-100	1
		5	3	>100	2

IMR: Index of Microcirculatory Resistance.

Two-dimension transthoracic echocardiography was performed seven days after the procedure to evaluate the left ventricular function and remodeling for all patients.

### Statistical Analysis

The statistical analysis was conducted using the SPSS Software (Version 23.0, SPSS Inc., Chicago, Illinois, USA). Continuous variables were tested for normal distribution with the Kolmogorov-Smirnov's test. Normally distributed data were presented as mean ± standard deviation (SD) and compared by the Student t-test between groups. Non-normally distributed data were presented as median (First Quartile, Third Quartile) and compared by the Mann-Whitney U test. Categorical variables were reported as percentage and compared using the chi-square or the Fisher's exact test. The discriminatory performance of the built model was examined by the receiver operating characteristic (ROC) curve. The illustration of the scores’ ROC was conducted using the MedCalc Software (Version 15.2.2, Med Calc Software bvba, Ostend, Belgium). The area under the curve (AUC), cut-off value, sensitivity, specificity, and corresponding Youden Index of each ROC was then obtained (Youden Index = sensitivity+ specificity -1). The comparison between scores was performed using a non-parametric test. A two-tailed *P*-value of <0.05 was considered statistically significant.

## Results

### Group Enrollment

From January 2018 to April 2018, a total of 65 eligible STEMI patients were enrolled in this study. Based on the final IMR threshold of 40, we allocated 48 patients in the NMO group and 17 in the MO group, with a CMVO incidence of 26.15%.

### Baseline Clinical Characteristics

The comparison of demographic data, baseline clinical characteristics, and preoperative laboratory tests between groups are shown in Table 3. No significant difference was observed in the following parameters: gender, body mass index (BMI), vital signs, previous history, red blood cell count, platelet count, high-sensitivity C-reactive protein (hs-CRP), electrolyte and lipid (All p>0.05). The mean age of the MO group was higher than that of the NMO group (p=0.002). The patients in the MO group shared a higher proportion of the Killip class 3 and a lower proportion of the Killip class 1. The GRACE and the CRUSADE scores were also significantly higher in the MO group. There were statistical differences in the following laboratory items between groups: white blood count, neutrophil count, lymphocyte count, neutrophil/lymphocyte ratio (NLR), CK-MB, cTNI, ACT, serum creatinine, eGFR, glucose, D-Dimer, and BNP (All p<0.05).

**Table 3:** Baseline clinical characteristics between groups

Variables		NMO group (n=48)	MO group (n=17)	p value
Age (years)		56.51±8.99	64.96±9.43	0.002
Male, n (%)		42(87.50)	13(76.47)	0.434
BMI (kg/m^2^)		24.76±3.31	25.52±3.12	0.412
Systolic blood pressure (mmHg)		128.54±19.30	136.67±22.49	0.158
Diastolic blood pressure (mmHg)		79.03±10.22	75.41±14.80	0.271
Heart Rate (bpm)		74.28±18.69	77.35±16.65	0.552
**Killip Grade**				
	Grade I, n (%)	29(60.42)	4(23.53)	0.009
	Grade II, n (%)	16(33.33)	4(23.53)	0.452
	Grade III, n (%)	3(6.25)	9(52.94)	<0.001
History of CAD, n (%)		25(52.08)	11(64.71)	0.368
Hypertension, n (%)		27(56.25)	10(58.82)	0.854
Diabetes, n (%)		15(31.25)	9(52.94)	0.111
Hyperlipidemia, n (%)		22(45.83)	8(47.06)	0.931
Smoking, n (%)		18(37.50)	9(52.94)	0.267
**Laboratory test on admission**				
	WBC count (10^9^/ L)	9.84±2.51	12.45±2.89	<0.001
Neutrophil count (10^9^/ L)		7.63(6.18, 9.09)	11.65(10.18, 13.00)	<0.001
Lymphocyte count (10^9^/ L)		1.60(1.26, 2.00)	1.46(1.08, 1.70)	0.184
N/L ratio		4.95(3.85, 7.00)	9.52(6.98, 10.56)	<0.001
hs-CRP (mg/L)		4.10(2.10, 6.55)	4.30(2.95, 7.30)	0.565
ACT		154(135, 178)	105(88, 132)	<0.001
CK-MB (U/L)		111 (43, 251)	168(84, 335)	0.044
Cardiac troponin I (ng/mL)		3.5 (1.8, 8.9)	14.0(6.0, 28.5)	<0.001
Serum Creatinine (μmol/L)		77.50(71.35, 86.15)	87.8 (77.5, 93.73)	0.038
Glomerular filtration rate (eGFR) (mL/min/1.73m^2^)		98.70±14.62	85.89±17.08	0.004
Serum Potassium (mmol/L)		3.81±0.55	3.83±0.43	0.886
LDL cholesterol (mmol/L)		2.87±0.67	2.80±0.83	0.717
Glucose (mmol/L)		8.57±1.88	11.31±2.41	<0.001
D-Dimer (μg/mL)		0.14(0.10, 0.23)	0.25 (0.16, 0.50)	<0.001
Type B natriuretic peptide, BNP (pg/mL)		50(26,150)	190(78,420)	0.003
**Preprocedural medication**				
Dual Antiplatelet Therapy, DAPT, n (%)		48(100.00)	15(88.24)	0.065
Statins, n (%)		24(50.00)	7(41.18)	0.531
Beta-blocker, n (%)		3(6.25)	2(11.76)	0.6
GRACE score		137.48±23.91	152.94±27.97	0.032
CRUSADE score		22.75±12.34	29.77±12.29	0.045

NMO: Non-microvascular obstruction; MO: microvascular obstruction; BMI: body mass index; CAD: coronary artery disease; hs-CRP: high-sensitivity C-reactive protein; ACT: activated clotting time;

### Angiographic Analysis and Invasive Measurement of Microvascular Perfusion

The angiographic features of all participants are summarized in Table 4. The SO-B time of the MO group was apparently delayed compared with that of the NMO group (p=0.002), while there wasn’t a significant difference in the FMC to FMC-B time (p=0.843). After the intervention, a significant difference regarding the blood flow perfusion indicators was observed, including TIMI 3 grade proportion (p<0.001), cTFC (p<0.001), and the proportion of TMPG 3 (p<0.001). Other angiographic and procedural information, such as IRA distribution, stenting details, medication, supplementary treatment, and contrast media volume were comparable between groups (All p>0.05).

**Table 4:** Procedural and angiographic features between groups

Variables		NMO group (n=48)	MO group (n=17)	p value
Onset to balloon (hours)		4.0(3.0, 5.0)	6.5(5.0, 12.0)	0.002
FMC to balloon (hours)		2.0(1.0, 3.0)	1.5(1.0, 2.8)	0.843
**Myocardial Wall, n (%)**				
	Anterior Wall	19(44.19)	9(52.94)	0.339
	Others	29(55.81)	8(47.06)	0.339
**Stenosed artery number, n (%)**				
1		9(18.75)	4(23.53)	0.729
	2	18(37.50)	7(41.18)	0.789
	3	21 (43.75)	6(35.29)	0.543
**Initial TIMI flow, n (%)**				
	0	27(56.25)	14(82.35)	0.055
	1	8(16.67)	2(11.76)	1
	2	8(16.67)	1(5.89)	0.426
	3	5(10.41)	0(0.00)	0.315
**Thrombus score, n (%)**				
	0-3	24(50.00)	1(5.56)	0.001
	4	20(41.67)	7(41.18)	0.972
	5	4(8.33)	9(53.26)	<0.001
**Final TIMI flow, n (%)**				
	0	0(0.00)	1(5.88)	0.262
	1	0(0.00)	3(17.65)	0.016
	2	0(0.00)	11(64.71)	<0.001
	3	48(100.00)	2(11.76)	<0.001
IRA-cTFC		24(20, 32)	48(36, 58)	<0.001
**TMPG, n (%)**				
	0	0(0.00)	2(11.76)	<0.001
	1	0(0.00)	5(29.41)	<0.001
	2	5(10.42)	11(58.83)	<0.001
	3	43(89.58)	0(0.00)	<0.001
**IMR-pre**				
	< 40	16(33.33)	1(5.58)	0.029
	40-100	20(41.67)	5(29.41)	0.372
	>100	12(25.00)	11(64.71)	0.003
**Stent number per patient, n (%)**				
1		42(87.50)	12(70.59)	0.138
	≤2	6(12.50)	5(29.41)	0.138
Stent length (mm)		23(21, 28)	24(18, 31)	0.143
Stent diameter (mm)		2.25(2.20, 3.00)	2.50(2.25, 3.00)	0.859
Pre-dilation pressure (atm)		14(12, 16)	14(12, 15)	0.307
Pre-dilation numbers		3(2, 5)	4(3, 5)	0.422
stent expansion pressure (atm)		14(14, 16)	14(12, 16)	0.347
Post-dilation pressure (atm)		16(12, 17)	14(11, 16)	0.776
Post-dilation numbers		2(2, 3)	2(1, 3)	0.689
Thrombus aspiration, n (%)		12(25.00)	3(17.64)	0.741
Temporary pacemaker, n (%)		4(8.33)	1(5.88)	1
Collateral circulation, n (%)		9(18.75)	3(17.65)	1
Contrast media volume (mL)		160(140, 190)	180(150, 210)	0.06
**Procedural medication, n (%)**				
	Tirofiban	43(89.58)	14(82.35)	0.421
	Bivalirudin	9(18.75)	5(29.41)	0.493
	Anisodamine	8(16.67)	3(17.65)	1

NMO: Non-microvascular obstruction; MO: microvascular obstruction; FMC: first medical contact; TIMI: thrombolysis in myocardial infarction; TMPG: TIMI myocardial perfusion grade.

### ROC Curve of Two Scores and Comparison of AUC

The corresponding scores of the two systems were calculated for all participants. Based on the scores and CMVO incidence, the ROC curve was plotted. For the SAK scores, the AUC was 0.855 [95% confidence interval (CI): 0.746 - 0.930], with a cut-off value of 15 and a Youden Index of 0.6078. For the ATI score, the AUC was 0.907 (95%CI: 0.809 - 0.965), with a cut-off value of 3 and a Youden Index of 0.6875. There was no significant difference in the AUC (Z=1.001, p=0.317) (Table 5).

**Table 5:** Comparison of AUC and related details of SAK and ATI Scores

Variables	AUC	95%CI	Cut-off point	Youden Index	Z	p
SAK Score	0.855	0.746 - 0.930	15	0.6078	1.001	0.317
ATI Score	0.907	0.809 - 0.965	3	0.6875		

### Echocardiography

All patients accepted transthoracic echocardiography after the procedure in the hospital. The left ventricular ejection fraction (LVEF) of the NMO group was higher than that of the MO group (56.03±5.22 vs. 47.79±6.38, p<0.001).

## Discussion

Despite the dramatic progress achieved in the therapeutic strategies of myocardial infarction in the past decades, microvascular impairment remains an important issue during primary catheterization. It is estimated that insufficient reperfusion in the myocardial tissue level could be up to 50% in cases, despite successful epicardial recanalization.^12^ The benefits resulting from pharmaceutical or mechanical reperfusion strategies would be compromised in the presence of coronary microvascular obstruction, which is associated with poor cardiac function and unfavorable outcomes.

Due to the lack of specific treatment and attenuation of CMVO, early recognition and the pretreatment of high-risk patients are of great importance. The indicators for identification have been intensively examined by sizable previous studies. However, considering that a large number of complicated mechanisms are thought to contribute to microvascular obstruction development, one single element may not be convincing enough in assessing risk prediction and stratification. Therefore, evaluating systems comprising of various indexes to assess the likelihood of this complication provides better detection and diagnosis. Apart from the two models analyzed in this study, previous scores of NFR have also been developed.

Dogan et al.^13^ reported that hyperglycemia, prolonged ischemic time, and low neutrophil count attributed to the development of the risk model.^13^ Bayramoglu et al.^14^ built the predictive model covering age, LVEF value, SYNTAX score, stent length, thrombus burden score, Killip classification, and reperfusion time.^14^ The retrospective study conducted by Wang et al.^15^ also showed that age, pain to PCI time, neutrophil count, admission glucose level, pre-PCI thrombus score, collateral circulation, and Killip class could be adopted to establish the no-reflow model.^15^ Due to the different study protocols, sample size, auxiliary measurements, and consistent conclusions have not been obtained.

Instead of angiographic standards (TIMI blood flow, TMPG or myocardial blush grades) applied in the former clinical trials, the IMR was introduced to determine the microcirculation perfusion in the present study. IMR, a thermodilution-derived quantitative measurement of coronary microvascular function, was first proposed by Fearon in 2003. The Porcine model has also been used to investigate the correlation between the calculated IMR value and true distal resistance, validating the feasibility of this innovative technique in estimating microvascular resistance.^6^ Different from other angiographically physiological and functional assessment, IMR shares the advantages of independence of epicardial stenosis, superior reproducibility, and hemodynamic instability. Bulluck reviewed the literature and reported that a post-procedure threshold of 40 U was valid in identifying CMVO for those that underwent IMR measurements.^16^

Apart from being directly related to the perfusion status of myocardial tissue, IMR was also shown to have a strong association with peak creatine kinase levels, patient's prognosis, and ventricular performance recovery in the setting of STEMI,^6^^,^^17^^–^^20^ which laid the foundation for ATI development. The ATI score was first introduced by De Maria et al. mainly consisting of three characteristics including age, thrombus score, and pre-stenting IMR value.,^5^ The ATI score was also considered a promising tool for predicting suboptimal myocardial reperfusion in STEMI patients and is correlated with the infarction area measured by cardiac magnetic resonance imaging (MRI) in subsequent studies.^21^

Limited by cost and related insurance regulations, however, IMR is not always available or acceptable in current practices. It could also only be implemented in the emergent Catheterization Laboratory. In this regard, based on the existing evidence and our practical experience, we systematically screened the possible clinical and angiographic information, developing SAK predictive scores that incorporate 6 conventional variables, namely age, Killip classification, symptom onset to balloon time, initial ACT levels, NLR, and glucose values. Our former study verified its capability and effectiveness in evaluating the patients at high risk of CMVO.^22^ Therefore, we attempted to compare the performance of the SAK and ATI scores in predicting the potential risk of impaired microvasculature during primary intervention, assisting the physicians’ prompt pretreatments to minimize the incidence of this condition before the procedure takes place.

Noticeably, an AUC or C-index value over 0.75 in a developed model is recognized as a reliable validation. From the results we obtained, the AUC of the SAK and ATI scores were 0.855 and 0.907, respectively, which proved that both estimating systems were capable of predicting the potential CMVO risk and performed well. The AUC of the ATI scores seemed higher, but there was no obvious difference in the risk evaluation performance.

Though the ATI score had a favorable performance for prediction, there were some distinctions compared to the original report of ATI development. Firstly, the most commonly chosen standard of the thrombus score was established by Gibson. However, according to clinical practice and previous data, only 0.4% of the cases had a score of 5 after the guidewire or balloon passing through the occluded lesions, whereas nearly 30% of the cases shared a score of 4.^23^ Consequently, the thrombus burden score was evaluated subsequent to the guidewire passing or small balloon inflation. Secondly, the peak value of myocardial biomarkers and cardiac troponin have not been documented as the original study, taking the echocardiography into consideration that the difference of the LVEF was also precise enough in showing the relationship between microcirculation perfusion and the infarction area. From the echocardiography results, we could derive that patients with CMVO had a poorer cardiac function, which was consistent with the existing evidence, emphasizing the particular significance in improving the perfusion status of microvascular circulation.^24^

Despite its advantages, the IMR is not available or applicable in a majority of local hospitals and many patients refuse this examination due to its cost. Similarly, a SAK score consisting of currently common indexes appeared to be an alternative in the clinical field.

This study, however, has some limitations. First, this was a single-center study with a relatively small sample size. The risk scores were validated by the information from a single-center database. The discriminatory power of the models requires a larger sample scale investigation and validation. Second, ACT was an essential element in the SAK score while the level of ACT is influenced by a series of factors in practice, so the reference range in the score might be different depending on the testing staff and equipment. Third, patients with cardiac shock have not been enrolled since supplementary life-supporting treatment might be needed and the baseline characteristics would be unbalanced for those patients.

## Conclusion

In this study, our data showed that both the SAK and ATI scores performed well in estimating CMVO risk after the primary PCI for acute STEMI patients. Therefore, these scores are accurate in predicting CMVO when compared to the invasive measurements obtained from the IMR.
